# Black Ginseng Extract Suppresses Airway Inflammation Induced by Cigarette Smoke and Lipopolysaccharides In Vivo

**DOI:** 10.3390/antiox11040679

**Published:** 2022-03-30

**Authors:** Mun-Ock Kim, Jae-Won Lee, Jae Kyoung Lee, Yu Na Song, Eun Sol Oh, Hyunju Ro, Dahye Yoon, Yun-Hwa Jeong, Ji-Yoon Park, Sung-Tae Hong, Hyung Won Ryu, Su Ui Lee, Dae Young Lee

**Affiliations:** 1 Natural Medicine Research Center, Korea Research Institute of Bioscience and Biotechnology (KRIBB), Cheongju 28116, Korea; mokim@kribb.re.kr (M.-O.K.); suc369@kribb.re.kr (J.-W.L.); alsrud5354@kribb.re.kr (Y.N.S.); zkx2@kribb.re.kr (E.S.O.); rh3cheld@kribb.re.kr (Y.-H.J.); parkgyun98@kribb.re.kr (J.-Y.P.); 2 Rpbio Research Institute, Rpbio Co., Ltd., Suwon 16229, Korea; ljk1200@rpcorp.co.kr; 3 Departments of Biological Sciences, College of Bioscience and Biotechnology, Chungnam National University, Daejeon 34134, Korea; rohyunju@cnu.ac.kr; 4 Department of Herbal Crop Research, National Institute of Horticultural and Herbal Science, Rural Development Administration, Eumseong 27709, Korea; dahyeyoon@korea.kr; 5 Departments of Anatomy & Cell Biology, Department of Medical Science, College of Medicine, Chungnam National University, Daejeon 35015, Korea; mogwai@cnu.ac.kr

**Keywords:** airway inflammation, black ginseng, cigarette smoke, Mucin 5AC, reactive oxygen species

## Abstract

Cigarette smoke (CS) is a risk factor that can induce airway enlargement, airway obstruction, and airway mucus hypersecretion. Although studies have shown that Korean black ginseng extract (BGE) has potent anti-inflammatory and antioxidant activities, the CS-induced inflammatory responses and molecular mechanisms are yet to be examined. The aim of this study was to examine the effect of BGE on the airway inflammatory response and its molecular mechanisms, using CS/lipopolysaccharides (LPS)-exposed animals and PMA-stimulated human airway epithelial NCI-H292 cells. The results show that BGE inhibited the recruitment of immune cells and the release of inflammatory mediators, such as tumor necrosis factor (TNF)-α and interleukin (IL)-6, monocyte chemoattractant protein (MCP)-1, elastase, and reactive oxygen species (ROS) in the airways of CS/LPS-exposed animals. BGE inhibited mucus secretion and the expression of Mucin 5AC (MUC5AC). Furthermore, BGE exhibited an anti-inflammatory effect by downregulating a signaling pathway mediated by transforming growth factor-β-activated kinase (TAK) 1, an important protein that accelerates inflammation by cigarette smoke (CS). Overall, the findings show that BGE inhibits lung inflammation and mucus secretion by decreasing the activation of TAK1 both in human epithelial cells and in CS/LPS-exposed animals, and could be a potential adjuvant in the treatment and prevention of airway inflammatory diseases caused by airway irritants such as CS.

## 1. Introduction

Ginseng (*Panax ginseng* C.A. Meyer) has been used as a tonic and herbal medicine in East Asia, including in China, Japan, and Korea [1]. Studies have shown that the major constituents (ginsenosides) of ginseng are effective therapeutic agents for inflammatory lung conditions [2,3,4]. Moreover, the therapeutic potential of ginseng extract for managing respiratory inflammatory disorders has been examined [5]. Raw ginseng can be processed into fresh ginseng, white ginseng, red ginseng, and black ginseng (BG) to improve its shelf-life and efficacy, using different technologies [6]. BG is a new type of the Korean ginseng prepared from fresh ginseng with over three steaming and drying cycles, which changes the ginseng color to black [7]. This repeated heating process improves its antioxidant activity, so the content of polar ginsenosides is relatively low compared to white and red ginseng [8]. Thus, the black ginseng extract (BGE) exhibits protective effects against lung inflammation and injury [9]. However, the molecular mechanisms of BGE in treating airway inflammatory response and mucus secretion, which can be triggered by smoke, are poorly understood.

Cigarette smoke (CS) is a primary risk factor that promotes the occurrence and development of pulmonary inflammation [10,11]. CS induces the release of inflammatory mediators such as interleukin-6 (IL-6), tumor necrosis factor-α (TNF-α), monocyte chemoattractant protein-1 (MCP-1), reactive oxygen species (ROS), and mucin (especially MUC5AC) in the lung tissue of patients with airway inflammatory disease [12,13]. Moreover, there are high levels of inflammatory cells and inflammatory mediators in bronchoalveolar lavage (BAL) fluids of smokers [14,15]. Inhalation of CS and lipopolysaccharides (LPS) in mice may accelerate inflammatory responses in lungs similar to those seen in patients with airway inflammatory disease [14,16,17]. These accumulated observations have led to the development of lung inflammatory mouse models, facilitating research into therapeutic agents for the treatment of patients with lung inflammatory disease [18].

Studies have shown that CS has the potential to activate mitogen-activated protein kinases (MAPKs) signaling and its downstream inflammatory transcription factors, such as cAMP response element-binding protein (CREB), early growth response gene (EGR) 1, and activator protein-1 (AP-1) [19,20,21]. MAPKs are three major serine/threonine kinases: extracellular receptor kinase-1/2 (ERK1/2), c-jun N-terminal kinase (JNK), and p38 MAPK, which have all been closely implicated in the pathogenesis of lung inflammatory disease [18]. These MAPKs cascades have been implicated in signal transduction pathways related to inflammatory mediators, mucus hypersecretion and immune cell infiltration, which are observed in patients with lung inflammatory disease [22]. Among the downstream molecules, ERK1/2 induces the secretion of inflammatory mediators, such as ROS, IL-6, and TNF-α through the activation of EGR1 and RSK/CREB [23,24]. JNK and p38 induce inflammation to allergens through activation of transcription factors including AP-1 (a heterodimeric protein of c-jun and c-fos) and CREB, which regulate cytokines and chemokines involved in the pathophysiology of pulmonary inflammatory disease [25,26]. Additionally, CS induces MUC5AC secretion by activating JNK, which is triggered by ROS [13]. Therefore, drugs or natural compounds that regulate MAPKs signaling and its downstream molecules could be potential therapeutic candidates for managing pulmonary inflammatory diseases symptoms.

Transforming growth factor-β-activated kinase 1 (TAK1, also called as MAP3K7) is a member of the mitogen-activated protein kinase (MAP3K) family that is a common upstream regulator of MAPKs signaling [27,28]. CS induces TAK1 activation, resulting in inflammatory responses involved in the development and progression of airway inflammatory diseases [29]. The selective inhibitor of TAK1, 5z-7-Oxozeaenol (5-OZ), reduces inflammatory response by suppressing of MAPK activities [29,30]. Therefore, TAK1 and its downstream MAPK signaling represent a major therapeutic target for the treatment of inflammatory respiratory diseases caused by airway irritants such as CS.

The aim of this study was to examine the anti-inflammatory activities of BGE processed from fresh ginseng and its molecular mechanism, using CS/LPS-exposed animals and PMA-stimulated human lung epithelial NCI-H292 cells.

## 2. Materials and Methods

### 2.1. Preparation of Black Ginseng Extract (BGE)

Panax ginseng was harvested to produce black ginseng from Eumseong, Chungbuk Province, Korea. The voucher specimen (RDA21-01) was deposited in the herbarium of the Department of Herbal Crop Research, National Institute of Horticultural and Herbal Science (NIHHS), RDA, Republic of Korea. Five-year-old roots were washed and dried by hot air. The dried roots were steamed at 95 ± 2 °C for 6 h and dried at 40 °C for 8 h, and this was repeated four times. The black ginseng produced was dried and homogenized. A powdered sample was extracted twice by reflux extraction with 95% ethanol (http://dhelife.co.kr/ accessed on 25 February 2022) and water at 80 °C for 4 h, filtered using a filter paper, and concentrated to 10.8 brix by a vacuum evaporator (Eyela, Tokyo, Japan). The product was dried in a spray dryer to produce dried extracts (BGE). For bulk production, the BGE manufacturing process was optimized and analyzed using HPLC. The sum of ginsenoside Rg3, Rg5, and Rk1 was 7.48 mg/g in BGE. The representative HPLC profiling chromatogram for BGE is presented in Figure S1.

### 2.2. Chemical and Reagents

Research cigarette 3R4F was acquired from the Tobacco and Health Research Institute (University of Kentucky, Lexington, KY, USA). Phorbol 12-myristate 13-acetate (PMA), lipopolysaccharide (LPS, from *E. coli* serotype 0111:B4), roflumilast (ROF), and 5z-7-Oxozeaenol (5-OZ) were purchased from Sigma-Aldrich (St. Louis, MO, USA). Anti-phospho(p)-TAK1, TAK1 -p-p38, -EGR1, -RSK, -p-CREB, -CREB, -c-jun, -ERK, -JNK and -Lamin-B1 antibodies were obtained from Cell Signaling Technology (Beverly, MA, USA). Anti-p-JNK, -p-c-jun, -p-ERK, -p-RSK, -p38 and -β-actin antibodies were acquired from Santa Cruz Biotechnology (Santa Cruz, CA, USA). RPMI 1640 was purchased from WELGENE Inc. (Gyeongsan-si, Republic of Korea). Fetal bovine serum (FBS), 100 μg/mL streptomycin 100 units/mL plus penicillin, and Trypsin-EDTA were purchased from Thermo Fisher Scientific (Waltham, MA, USA).

### 2.3. CS Exposure and LPS Treatment to Generate Lung Inflammation Mice Model

Six-week-old male C57BL/6 mice (20–25 g, Koatech Co., Pyeongtaek, Republic of Korea) were whole-body exposed to fresh air or CS from 8 cigarettes for 1 h per day for 7 days using a smoking machine (SciTech Korea, Inc., Seoul, Korea). LPS (5 μg dissolved in 50 μL distilled water) was intranasally administered on day 6. BGE and roflumilast (ROF, inhibitor of phosphodiesterase 4) were orally administered to the animals 1 h before CS exposure for 7 days. ROF was used as a positive control agent because it is an approved drug for the treatment of chronic inflammatory disease [31]. For the experiment, the mice were randomly divided into six treatment groups (*n* = 6 per group): normal control (NC) group, CS and LPS exposure (CS/LSP) group, CS/LSP + 5 mg/kg ROF group, CS/LSP + 25 mg/kg BGE group, CS/LSP + 50 mg/kg BGE group, and CS/LSP + 100 mg/kg BGE group.

### 2.4. Analysis of Bronchoalveolar Lavage (BAL) Fluid of Animal Model

After anesthetizing the mice with xylazine and Zoletil 50^®^ on day 8, BAL fluid sampling and tracheostomy were performed as previously described [32]. ROS level was determined using 2′, 7′-dichlorofluorescin diacetate (DCF-DA, Sigma-Aldrich, Carlsbad, CA, USA), according to previously reported protocol [33]. Additionally, TNF-α, IL-6, and MCP-1 levels were determined using standard ELISA kits (BD Biosciences, San Jose, CA, USA), according to the manufacturer’s protocols. To calculate the number of inflammatory cells in BAL fluid samples, the samples were centrifuged using a CytoSpin 3 cytocentrifuge (Thermo Fisher Scientific, Waltham, MA, USA). Thereafter, the centrifuged preparations were stained on a slide using Diff-Quik^®^ reagent (SYSMEX, Kobe, Japan) and the inflammatory cells were counted using a light microscope at 400× magnification. The number of inflammatory celsl was determined as the average of counted cells in five different fields [16].

### 2.5. Histological Examination

For histological analysis, lung tissue was cleaned with PBS, fixed with 10% formalin, embedded in paraffin, and cut into 4 μm thick sections using a rotary microtome. Thereafter, the sections were stained with hematoxylin and eosin (H&E, Sigma-Aldrich St. Louis, MO, USA) and periodic acid-Schiff (PAS, IMEB, Inc., San Marcos, CA, USA) stain solution, according to the manufacturer’s instruction. Lung tissue samples were visualized under a light microscope (H&E staining: 100× magnification; scale bar, 100 μm; PAS staining: 400× magnification; scale bar, 50 μm).

### 2.6. Cell Maintenance

NCI-H292 cells, a human pulmonary muco-epidermoid carcinoma line, were purchased from the American Type Culture Collection (CRL-1848; ATCC, Manassas, VA, USA) and were used at early passages (7–20 passages) in all experiments. The cell culture was performed as previously described [34].

### 2.7. Cell Viability Assay

Cells were seeded on 96-well plates at a density of 8 × 10^3^ cells/well for 16 h. The medium was subsequently replaced with serum-reduced (0.1% FBS) RPMI-1640 medium. After 16 h incubation, the cells were treated with the indicated concentrations of BGE for 24 h. Cell growth was analyzed in triplicate using a Cell Counting Kit-8 (Dojindo Molecular Technologies, Rockville, ML, USA), according to the manufacturer’s protocol. Optical absorbance was determined using an Epoch microplate reader (BioTek instruments, Inc., Winooski, VT, USA) and converted to the percentage (%) of the control value.

### 2.8. Enzyme Linked Immunosorbent Assay (ELISA) Assay

Cells were seeded on 96-well plates at a density of 8 × 10^3^ cells/well for 16 h. The medium was subsequently replaced with RPMI-1640 medium with 0.1% FBS. The cells were pre-treated with BGE (20, 40, and 80 μg/mL) for 2 h and were then stimulated with PMA (100 nM) before collecting the supernatant; 7 h for TNF-α and IL-6, and 22 h for MUC5AC. The level of cytokines in the supernatant was determined using ELISA kits (BD Pharmingen, San Diego, CA, USA), according to the manufacturer’s instructions. The level of MUC5AC protein in the cell culture supernatant was determined as previously described [35,36], and the absorbance of the solution was measured at 450 nm using an Epoch microplate reader (BioTek instruments, Inc., Winooski, VT, USA).

### 2.9. Western Blot Analysis

Cells (2 × 10^5^ cells/well) were seeded in 6-well plates, and cultured in growth medium for 16 h, and subsequently replaced with a medium containing 0.1% serum. After 16 h of culture, the cells were pre-treated with the indicated concentrations of BGE or CK for 2 h and then treated with 1 μM PMA for 30 min. At least 30 μg whole cell lysate or 10 μg nucleus fraction was prepared and loaded as described previously [34]. Protein bands were visualized using a LAS-4000 luminescent image analyzer (Fujifilm, Tokyo, Japan) and quantified using densitometry (Fuji Multi Gauge software version 3.0). The lung tissue lysate was homogenized in a RIPA lysis buffer (1/10 *w*/*v*) containing protease inhibitor cocktail (Sigma, St. Louis, MO, USA) [16].

### 2.10. TAK1 Kinase Activity

TAK1 kinase activity in vitro was determined using a TAK1 kinase enzyme system kit (Promega Corporation, WI, USA). Briefly, aliquots of a mixture of TAK1 protein (0.1 mg/mL) diluted in reaction buffer A and MBP (native swine myelin basic protein) substrate (0.1 μg/μL) were transferred into a 96-well white plate. Then, the mixture was incubated at 30 °C for 10 min in the presence or absence of compounds. After adding ATP, the plate was stored at 30 °C for 15 min, and ADP-Glo reagent was added for 40 min to terminate the kinase reaction and remove the remaining ATP. The kinase detection reagent was added to the mixture and the plate was shaken for 25 min, and the luminescence was measured using SpectreMax M4 microplate reader (Molecular Devices, CA, USA).

### 2.11. Ethics Statement

The procedures for the animal experiments were approved by the IACUC on 10 April 2020 by the Korea Research Institute of Bioscience and Biotechnology (KRIBB, Chungbuk, Korea; KRIBB-AEC-20216). The procedure was also performed in compliance with the NIH Guidelines for the Care and Use of Laboratory Animals, as well as the Korean national laws for animal welfare.

### 2.12. Statistical Analysis

Data are presented as mean ± standard deviation (SD). Statistical significance was analyzed using a two-tailed Student’s *t*-test for in vitro experiments. Statistical significance was set at * *p* < 0.05, ** *p* < 0.01, and *** *p* < 0.001. One-way analysis of variance (ANOVA) followed by Tukey’s multiple comparison test were used to analyze data from in vivo experiments, using SPSS (version 20.0; IBM Corp., Armonk, NY, USA). Single asterisks (*) represent statistical significance at *p* < 0.05.

## 3. Results

### 3.1. BGE Reduces the Secretion of Inflammatory Mediators in Lung Inflammation Mice Model

**Figure 1:**
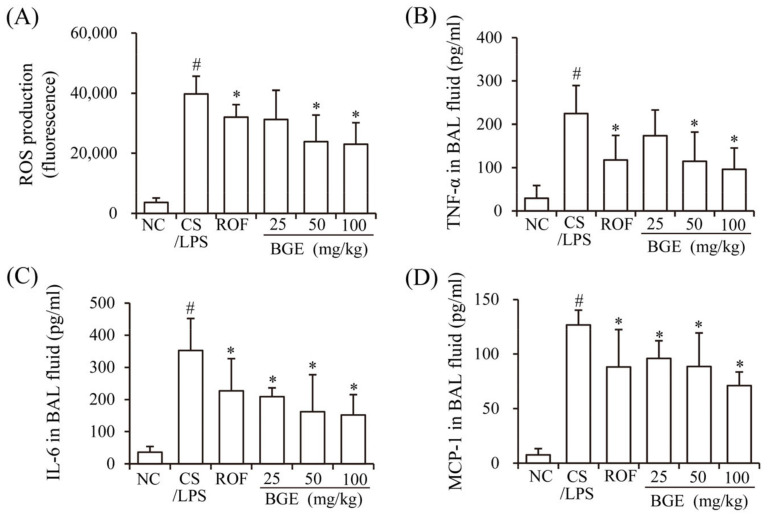
Effects of the black ginseng extract (BGE) on airway inflammatory responses in in CS/LPS-treated lung inflammation mice model. (**A**) ROS level of bronchoalveolar lavage (BAL) fluid was determined using 2′, 7′-dichlorofluorescin diacetate (DCF-DA); (**B**–**D**) The TNF-α, IL-6, and MCP-1 levels of BAL fluid were determined using ELISA kits. Roflumilast (ROF) was used as a positive control agent. NC: normal control mice; CS/LPS: CS/LPS-treated mice; ROF: 5 mg/kg ROF-treated CS/LPS mice; BGE 25: 25 mg/kg BGE-treated CS/LPS mice; BGE 50: 50 mg/kg BGE-treated CS/LPS mice; and BGE 100: 100 mg/kg BGE-treated CS/LPS mice group. The values are expressed as means ± standard deviation (SD) (*n* = 6). # *p* < 0.01: significantly different compared with NC; * *p* < 0.05: significantly different compared with CS/LPS-induced group.

Previously, CS has been shown to induce the production of inflammatory mediators, such as ROS, cytokines (TNF-α and IL-6), and chemokine (MCP-1), resulting in lung inflammation [15]. LPS accelerates inflammatory responses in vivo [17] similar to that of chronic lung inflammatory disease in humans [14]. Therefore, we investigated whether BGE administration can ameliorate CS/LPS-induced airway inflammatory responses in mice. The results show that there was a marked increase in the ROS, TNF-α, IL-6, and MCP-1 levels of the BAL fluids of CS/LPS-treated mice compared with those of mice in the NC group (Figure 1A–D). However, BGE and ROF treatments significantly suppressed the secretion of the inflammatory factors and ROS in CS/LPS-exposed mice. ROF, a FDA-approved chronic lung inflammatory disease medication, was used as a positive control [31]. These results indicate that BGE possesses anti-inflammatory activity in the CS/LPS-treated lung inflammation mice model.

### 3.2. BGE Reduces Recruitment of Inflammatory Cells and Mucus Secretion in Lung Inflammation Mice Model

**Figure 2:**
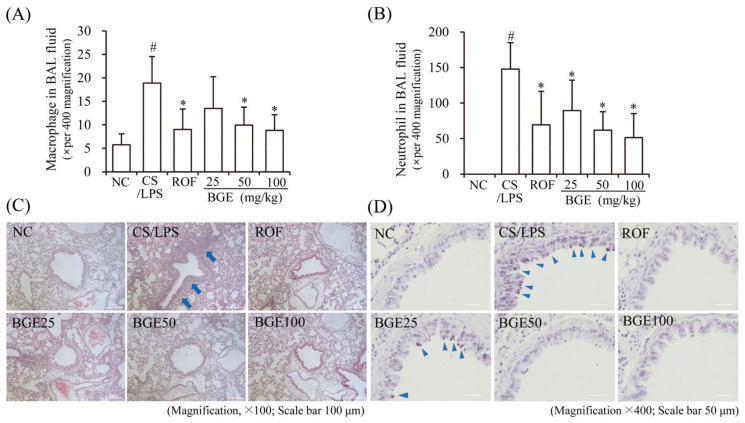
Effects of the BGE on the recruitment of inflammatory cells and secretion of mucus in the airway of the CS/LPS-treated mice model. (**A**,**B**) Cells were isolated from BAL fluids and stained with Diff-Quik reagent. The values are expressed as means ± standard deviation (SD) (*n* = 6). # *p* < 0.01: significantly different compared with NC; * *p* < 0.05: significantly different compared with CS/LPS -induced group. (**C**) Hematoxylin and eosin (H&E) staining was performed to assess the accumulation of inflammatory cells around the peribronchial region. Blue arrows indicate regions where inflammatory cells are concentrated. Magnification, 100×, scale bar, 100 μm. (**D**) Mucus secretion level in the lung tissue was determined using periodic acid-Schiff (PAS) staining. Blue arrowheads indicate mucus. Magnification, 400×, scale bar, 50 μm. ROF was used as a positive control. NC: normal control mice; CS/LPS: CS/LPS-exposed mice; ROF: 5 mg/kg ROF-treated CS/LPS mice; BGE 25: 25 mg/kg BGE-treated CS/LPS mice; BGE 50: 50 mg/kg BGE-treated CS/LPS mice; and BGE 100: 100 mg/kg BGE-treated CS/LPS mice group. Representative images were randomly selected from among 3 to 4 images.

CS has been shown to increase the proliferation and recruitment of immune cells, such as neutrophils and macrophages in BAL fluid and lung tissue in vivo [15]; therefore, we examined the effect of BGE administration on the proliferation of inflammatory cells in CS/LPS-treated mice. The results show that there was an increase in the accumulation of inflammatory cells, including neutrophils and macrophages, in the lung tissue of CS/LPS-treated mice; however, ROF and BGE significantly suppressed the CS/LPS-induced increase in the proliferation of inflammatory cells (Figure 2A,B). Additionally, H&E staining indicated the recruitment of inflammatory cells to the lung with dense peribronchial infiltrates in CS/LPS-treated mice (Figure 2C, blue arrow); however, this histopathological change was significantly suppressed by BGE treatment. Furthermore, PAS staining showed increased mucus production in the lung tissue epithelium in the CS/LPS-induced group (Figure 2D, triangular blue arrow), which was inhibited by BGE treatment. These data indicate that BGE could ameliorate the lung inflammatory responses by suppressing inflammatory responses and mucus secretion in the CS/LPS-treated mice model.

### 3.3. BGE Suppresses the Secretion of Inflammatory Cytokines and MUC5AC in PMA-Stimulated NCI-H292 Cells

**Figure 3:**
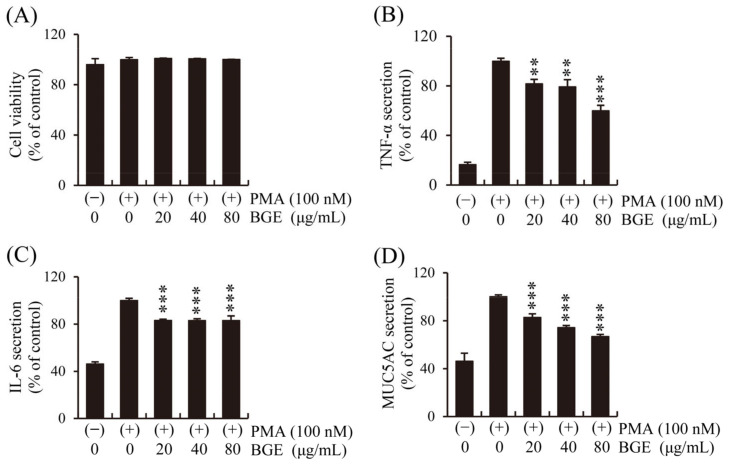
Effects of the BGE on PMA-induced increase in TNF-α, IL-6, and MUC5AC secretion in human airway epithelial (NCI-H292) cells. Cells were pre-treated with the corresponding concentration for 2 h and subsequently exposed to PMA (100 nM) for 16 h. (**A**) NCI-H292 cell viability was assessed using the CCK-8 method. (**B**–**D**) TNF-α, IL-6, and MUC5AC levels were determined using ELISA kits. Bar graphs represent means ± S.D. in triplicate in one representative experiment from three independent experiments (** *p* < 0.01, and *** *p* < 0.001 for comparison with controls, which were treated with PMA alone).

Human lung epithelial cells initiate the first immune response to CS, and epithelial changes such as abnormal proliferation of goblet cells in the airways are important characteristics of pulmonary inflammatory disease [37]. First, the effect of BGE and PMA on NCI-H292 cell viability was performed. The CCK-8 assay showed that BGE had no significant cytotoxicity at concentrations below 80 μg/mL (Figure 3A). Therefore, the effect of BGE (<80 μg/mL) on inflammatory cytokines secretion by human lung epithelial cells was examined using ELISA kits, and the results show that BGE suppressed PMA-induced increase in TNF-α, IL-6, and MUC5AC levels in a dose-dependent manner (Figure 3B–D). These results suggest that BGE possesses anti-inflammatory activity in PMA-stimulated NCI-H292 cells.

### 3.4. BGE Inhibits PMA-Stimulated TAK1 and Its Downstream Targets in NCI-H292 Cells

**Figure 4:**
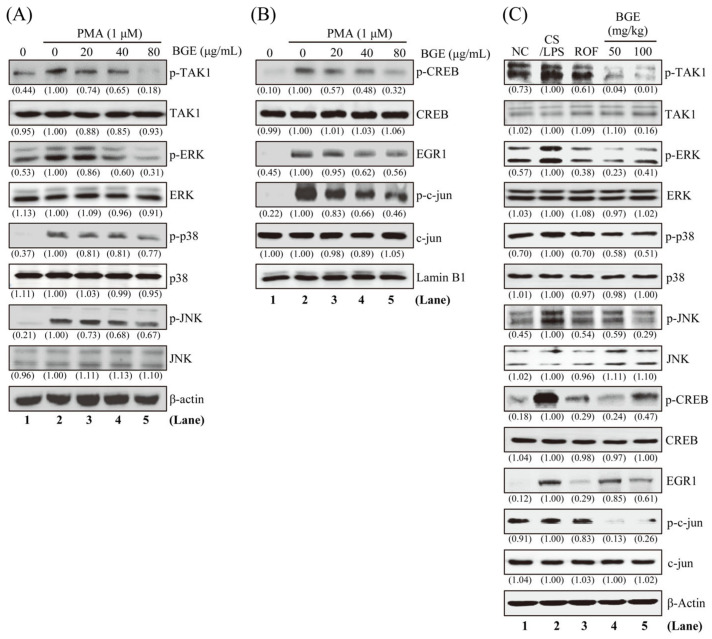
The effects of the BGE on TAK1 and MAPK signaling pathways in NCI-H292 cells and lung tissues of the CS/LPS-treated mice model. (**A**,**B**) Cells were pre-treated with indicated concentrations of BGE for 2 h, and subsequently treated with 1 μM PMA for 30 min. Total cell lysates (**A**) or nuclear extracts (**B**) were assayed by Western blotting using antibodies against p-TAK1, TAK1, p-ERK, ERK, p-JNK, JNK, p-p38, p38, p-CREB, CREB, EGR1, p-c-jun, and c-jun. β-actin or Lamin B1 were used as a loading control for the total lysates and nuclear extracts, respectively. The numbers underneath the bands indicate the relative band intensity to the PMA-treated control. (**C**) Western blot analysis was performed to detect the activation of TAK1 and its downstream molecules in the lung tissues of CS/LPS-treated mice using antibodies against p-TAK1, p-ERK, p-JNK, p-p38, p-CREB, EGR1, and p-c-jun. β-actin was used as a loading control for the total lysates of lung tissues. The lane numbers are indicated below the Western blot image. The numbers underneath the bands indicate the relative band intensity to control which were treated with CS/LPS alone. Western blotting was independently performed twice. NC: normal control mice; CS/LPS: CS/LPS-exposed mice; ROF: 5 mg/kg ROF-treated CS/LPS mice; BGE 25: 25 mg/kg BGE-treated CS/LPS mice; BGE 50: 50 mg/kg BGE-treated CS/LPS mice; and BGE 100: 100 mg/kg BGE-treated CS/LPS mice group.

Since TAK1 and its downstream MAPKs signaling are an important target for lung inflammatory disease treatment [28,29], we examined whether BGE can inhibit these molecules in NCI-H292 human lung epithelial cells. Western blot analysis was performed using antibodies that can detect the phosphorylated form of TAK1 and its downstream MAPKs. PMA increased the phosphorylation of TAK1 and three MAPKs (ERK, JNK, and p38); however, 80 μg/mL BGE considerably inhibited the activation of these molecules without change in protein expression in NCI-H292 cells (Figure 4A and Figure S2A). Additionally, BGE was more effective in inhibiting the phosphorylation of TAK1 and ERK than p38 and JNK phosphorylation. Next, we evaluated whether BGE treatment affected the phosphorylation of downstream transcription factors of ERK, such as EGR1 and CREB in the nucleus. Figure 4B shows that BGE suppressed PMA-induced phosphorylation of EGR1 and CREB in the nucleus. Moreover, BGE inhibited the phosphorylation of c-jun, a component of AP-1, in the nucleus (Supplementary Figure S2B). Additionally, BGE significantly inhibited TAK1 and its downstream signaling molecules in the lung tissue of CS/LPS-treated mice (Figure 4C and Figure S2C). Generally, CS/LPS treatment increased TAK1, MAPKs, CREB, EGR1, and c-jun phosphorylation; however, BGE and ROF inhibited the phosphorylation of these molecules. ROF significantly inhibited MAPK phosphorylation, which was similar to previous results [16]. Collectively, the results suggest that BGE may exert anti-inflammatory effects by negatively regulating TAK1 and its downstream MAPKs signaling pathways in lung inflammatory disease mice models as well as in PMA-stimulated lung epithelial cells.

### 3.5. BGE Directly Inhibits TAK1 Kinase Activity

**Figure 5:**
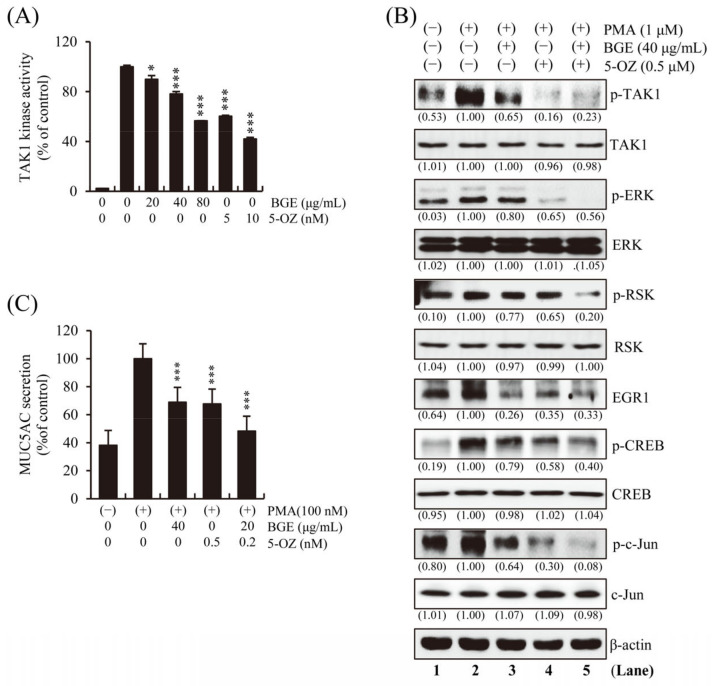
Effects of BGE on TAKI activity in NCI-H292 cells. (**A**) In vitro TAK1 enzymatic kinase assay was performed. Bar graphs represent means ± S.D. in triplicate in one representative experiment from three independent experiments (* *p* < 0.05 and *** *p* < 0.001 compared with TAK1 enzyme alone). (**B**) The cells were pre-treated with indicated concentrations of BGE and/or 5-OZ (a TAK1 inhibitor) for 2 h, and subsequently treated with PMA (1 μM) for 30 min. Total cell lysates were assayed by Western blotting using antibodies against p-TAK1, TAK1, p-ERK, ERK, p-RSK, RSK, p-CREB, CREB, EGR1, p-c-jun and c-jun. Β-actin was used as a loading control. The numbers underneath the bands indicate the relative band intensity to controls, which were treated with PMA alone. The lane numbers are indicated below the western blot image. (**C**) MUC5AC secretion was determined using ELISA kit. Cells were pre-treated with respective concentrations of the treatments for 2 h and subsequently exposed to PMA (100 nM) for 16 h. The bar graph presents means ± SD of three independent experiments (*** *p* < 0.001 compared with the PMA alone). Western blotting was independently performed twice.

To determine whether BGE directly inhibits the enzymatic activity of TAK1, an in vitro TAK1 enzymatic kinase assay was performed under cell-free conditions. The results show that TAK1 kinase activity was significantly inhibited by BGE in a dose-dependent manner (Figure 5A). A selective TAK1 inhibitor, 5z-7-Oxozeaenol (5-OZ), was used as a positive control. PMA-induced TAK1, ERK, RSK, CREB, and EGR1 phosphorylation was effectively suppressed by BGE + 5-OZ, which had the best effect compared with BGE or 5-OZ alone (Figure 5B and Figure S3). These results indicate that BGE could prevent the activation of MAPK and its downstream molecules by suppressing TAK1 activation. The fact that BGE or 5-OZ does not change the total protein expression of TAK1, ERK, RSK, CREB and c-jun within the experimental conditions confirms that TAK1 and its downstream kinase activity is inhibited. Additionally, the effect of BGE on MUC5AC secretion was examined, and the results show that BGE + 5-OZ was more effective than BGE or 5-OZ alone in inhibiting MUC5AC secretion (Figure 5C).

**Figure 6:**
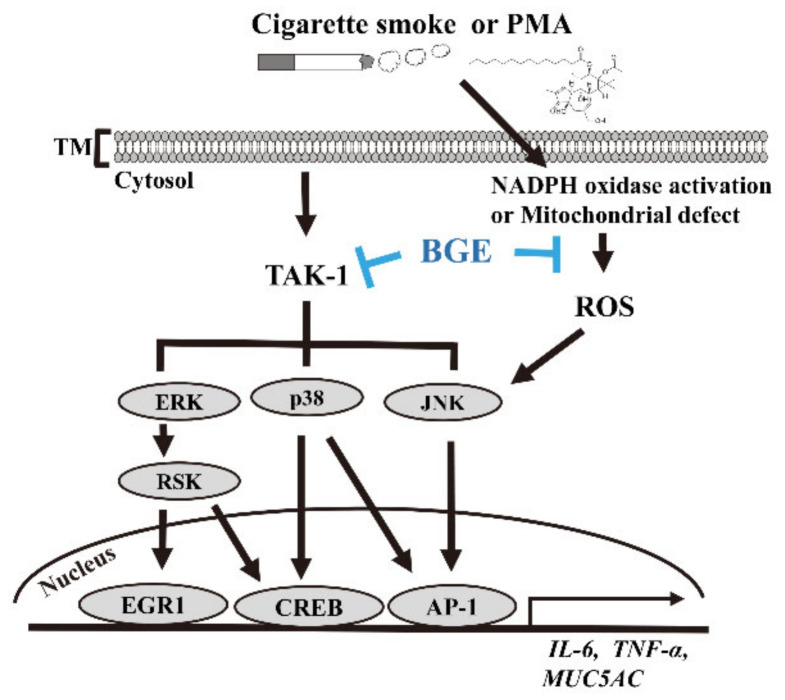
The anti-inflammatory mechanism of BGE in both lung airway epithelial cells and the CS/LPS-treated mice model. CS or PMA activates TAK1 and its downstream molecule MAPK signaling. This phosphorylation of MAPKs eventually results in a series of inflammatory reactions in lung epithelial cells and in the CS/LPS-treated mice model. BGE suppresses the expression of inflammatory cytokines, such as TNF-α, IL-6 and MUC5AC (the major form of secreted gel-forming mucin in vivo), both in vivo and in vitro. BGE directly binds to TAK1, inhibiting its activity. BGE exerts its anti-inflammatory effect by inhibiting the phosphorylation/activation of three MAPKs by directly suppressing TAK1 activity, leading to inactivation of inflammatory transcription factors, such as EGR1, CREB and AP-1.

This result indicates that BGE could downregulate TAK1 and downstream target genes, such as MUC5AC. Overall, these results confirm that BGE could ameliorate airway inflammation and mucus secretion by suppressing TAKI, and consequently MAPK and its downstream signaling (Figure 6).

## 4. Discussion

BG is a type of processed ginseng obtained by repeatedly steaming and drying fresh ginseng. This process leads to extensive changes in the type and amount of secondary metabolites. The main secondary metabolite is ginsenoside, which is changed to less polar ginsenoside by the steaming and drying process. Additionally, other secondary metabolites are affected by the processing. The content of phenols, reducing sugars and acidic polysaccharides increases, and the content of free amino acids and total polysaccharides decreases [38,39]. These chemical changes are related to the marked superiority of BG over white and red ginseng in most comparative biological studies [40,41].

Although a recent study examined the anti-inflammatory effect of processed ginseng on lung inflammation caused by fine particulate matters [17], the potential therapeutic mechanism of BGE in CS-related lung inflammation is poorly understood. In this study, the anti-inflammatory molecular mechanism of BGE on lung inflammation was examined using PMA-stimulated lung epithelial cells and a CS/LPS-treated lung inflammation mice model.

CS has been recognized as a major risk factor for pulmonary inflammatory disease, because it can induce the secretion of inflammatory mediators and recruitment of immune cells [15]. Studies have shown increased levels of inflammatory mediators, such as cytokines (TNF-α and IL-6), chemokines (MCP-1 and IL-8), ROS, and mucus in the airway of cigarette-smokers and patients with chronic inflammatory disease [16,24]. The findings of the present study show that BGE effectively decreased secretion of mucus, the infiltration of immune cells, and inflammatory mediators such as cytokines (TNF-α and IL-6), chemokine (MCP-1) and ROS in the lung tissue of CS/LPS-exposed model mice. Interestingly, the anti-inflammatory activity of BGE in the lung tissues of CS/LPS-exposed mice was comparable to that of ROF, a drug for treating chronic inflammatory patients [31]. Furthermore, consistent with in vivo results, BGE inhibited the secretion of inflammatory mediators including MUC5AC, TNF-α, IL-6 and IL-8 in PMA-stimulated NCI-H292 cells. These findings suggest that BGE could be used to manage lung inflammatory diseases, which are induced by airway irritants such as CS. CS has been shown to induce activation of TAK1 and its downstream MAPKs (ERK1/2, JNK and p38), which are the major signaling axis in pathogenesis of lung inflammatory diseases [42]. MAPKs signaling regulates the expression of inflammatory genes by promoting the activation of inflammatory transcription factors, such as EGR1, AP-1 (a heterodimeric protein of c-jun and c-fos), or CREB [23]. Thus, the TAK1/MAPKs axis is responsible for the regulation of CS-associated inflammatory responses and tissue damage. Therefore, natural compounds targeting TAK1 and MAPKs could be manage symptoms of lung inflammatory disease [43]. The findings of the present study show that BGE exerted its anti-inflammatory activity by inactivating downstream effectors, such as EGR1, AP-1 and CREB, by suppressing the activation of TAK1 and MAPKs in both PMA-stimulated human lung epithelial cells and in lung tissue of the CS/LPS-treated mice. Specifically, BGE suppressed TAK1 phosphorylation, an apical kinase regulating MAPKs, both in vitro and in vivo.

Further experiments confirmed that BGE directly suppressed the enzymatic activity of TAK1. Previous studies have shown that resveratrol (3,5,4’-trihydroxy-trans-stilbene), which directly targets TAK1, reduces the expression level of inflammatory cytokines and relieves symptoms in the CS/LPS-induced mice model, smokers, and patients with pulmonary inflammatory disease [44,45], indicating that direct inhibition of TAK1 improves the symptoms of lung respiratory disease [46]. Furthermore, a combination of BGE with 5-OZ (TAK1 inhibitor) synergistically suppressed the expression of inflammatory genes by inhibiting the activities of TAK1 and its downstream molecules. Moreover, a combination of BGE with 5-OZ synergistically inhibited MUC5AC secretion. These findings support that BGE can ameliorate airway inflammation and mucus hypersecretion by inhibiting the activities of TAK1 and apical signaling kinases of three MAPKs.

## 5. Conclusions

Overall, we showed that BGE exert anti-inflammatory activity both in vitro and in vivo by directly inhibiting TAK1 activity, thereby suppressing MAPKs and their downstream transcription factors (EGR1, CREB, and AP-1). BGE effectively inhibited the inflammatory response caused by CS in our results. There is potential for BGE to be used as a supplement for smokers or people with risk factors for respiratory disorders caused by air pollution or irritants. Therefore, we suggest that BGE could be a promising material for treating lung inflammatory diseases.

## Data Availability

Data is contained within the article or Supplementary Materials.

## Supplementary Materials

The following supporting information can be downloaded at: https://www.mdpi.com/article/10.3390/antiox11040679/s1. Figure S1: (A) Representative HPLC chromatogram of Black Ginseng Extract (BGE), (B) Overlay chromatogram of ginsenosides. Figure S2: Raw data from Western blot in Figure 4. Figure S3: Raw data from Western blot in Figure 5. Figure S4: Quantification and Statistical Analysis of Western Blot in Figure 4. Figure S5: Quantification and Statistical Analysis of Western Blot in Figure 5.
