# Supplementary material for: Black Ginseng Extract Suppresses Airway Inflammation Induced by Cigarette Smoke and Lipopolysaccharides In Vivo
Source: Antioxidants (Basel). 2022 Mar 30;11(4):679. doi: 10.3390/antiox11040679 (PMC9025275; doi:10.3390/antiox11040679)
Supplement: Supplementary file 1 [file antioxidants-11-00679-s001.zip › antioxidants-1640310-supplementary.pdf]

Supplementary Information

Black Ginseng Extract Suppresses Airway Inflammation in-duced by cigarette smoke and lipopolysaccharides *in vivo*

Mun Ock Kim *et al.*,

• Supplementary Figures

Figure S1. (A) Representative HPLC chromatogram of Black Ginseng Extract (BGE), (B) overlay chromatogram of ginsenosides 2

Figure S2. Raw data from western blot in Figure 4 3

Figure S3. Raw data from western blot in Figure 5 3

Figure S4. Quantification and Statistical Analysis of Western Blot in Figure 4 4

Figure S5. Quantification and Statistical Analysis of Western Blot in Figure 5 4

Experimental Section

Western blot analysis and quantification

Western blots were performed as described in the "Materials and Methods" section of this document. The results of three independent experiments were quantified using densitometry (Fuji Multi Gauge software version 3.0), and the relative ratio compared to the control group (PMA alone) was graphed.

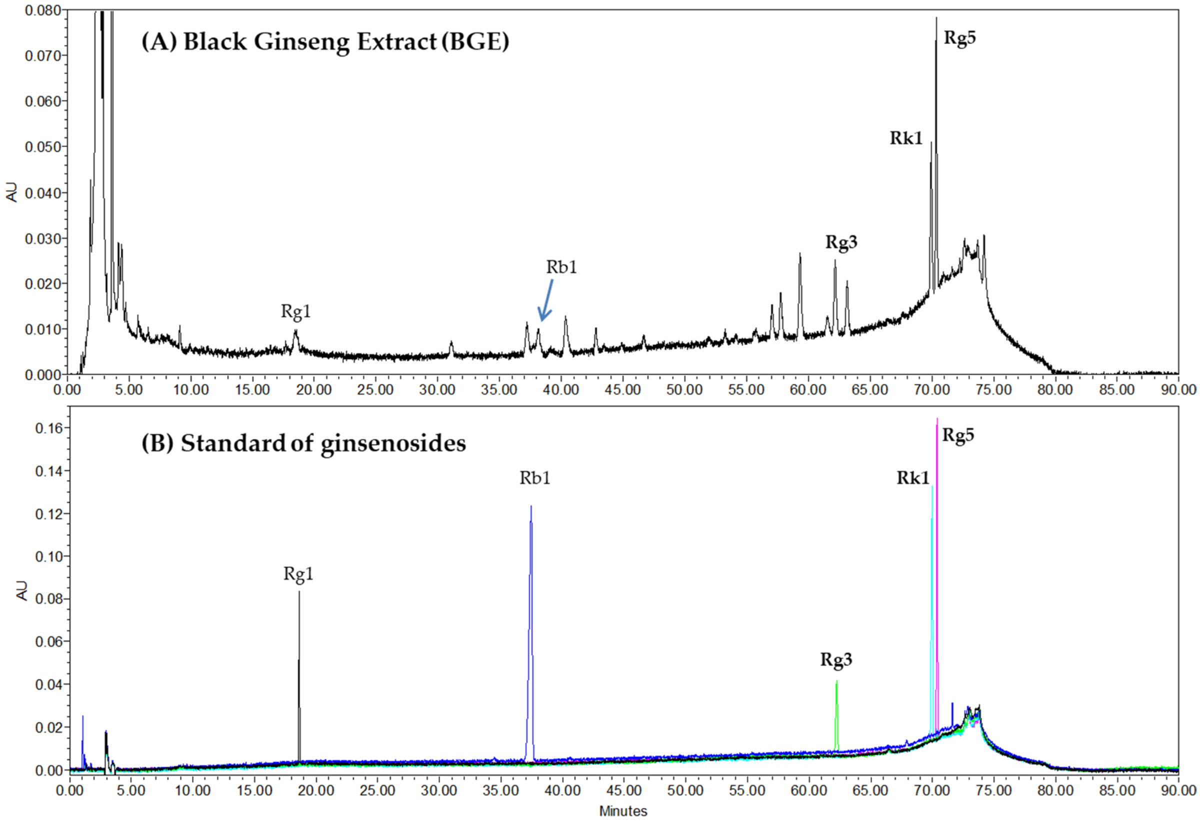

【 LC conditions 】

① Instrument

Instrument	
HPLC(HPLC-PDA)	[Waters] Alliance e2695 HPLC

② Solvents

Solvent	
Solvent A	D.W.
Solvent B	ACN

③ Method - Gradient

Time(min)	Flow (mL/min)	%A	%B
(Initial)	1.0	90	10
10	1.0	79	21
15	1.0	71	29
20	1.0	71	29
35	1.0	68	32
60	1.0	50	50
65	1.0	40	60
70	1.0	0	100
75	1.0	90	10

④ Column
YMC-Pack ODS-AM 5㎛ 4.6X250㎜ Column

⑤ inj. Volume: 10 μl

⑥ Absorbance
UV : 203 nm

Figure S1. (A) Representative HPLC chromatogram of Black Ginseng Extract (BGE), (B) overlay chromatogram of ginsenosides.

A

Target	Size	#1	#2
p-TAK1	82 kDa		
TAK1	82 kDa		
p-ERK	42/44 kDa		
ERK	42/44 kDa		
p-p38	38 kDa		
p38	38 kDa		
p-JNK	46/54 kDa		
JNK	46/54 kDa		
Actin	43 kDa		

B

Target	Size	#1	#2
p-CREB	43 kDa		
CREB	43 kDa		
p-c-jun	48 kDa		
c-jun	48 kDa		
EGR1	75 kDa		
Lamin B1	67 kDa		

C

Target	Size	#1	#2
p-TAK1	82 kDa		
TAK1	82 kDa		
p-ERK	42/44 kDa		
ERK	42/44 kDa		
p-JNK	46/54 kDa		
JNK	46/54 kDa		
p-p38	38 kDa		
p38	38 kDa		
p-c-jun	39 kDa		
c-jun	39 kDa		
p-CREB	43 kDa		
CREB	43 kDa		
EGR1	75 kDa		
Actin	43 kDa		

Figure S2. Raw data from western blot in Figure 4. The number of each independent experiment is indicated by #1 and #2.

Target	Size	#1	#2
p-TAK1	82 kDa	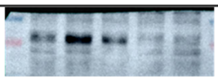	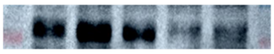
TAK1	82 kDa	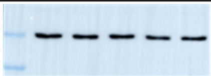	
p-ERK	42/44 kDa	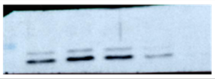	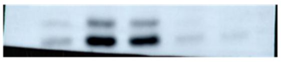
ERK	42/44 kDa	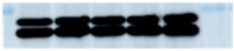	
p-RSK	90 kDa	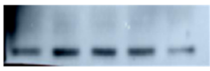	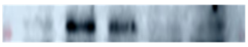
RSK	90 kDa	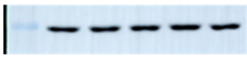	
p-CREB	43 kDa	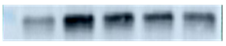	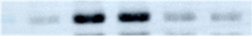
CREB	43 kDa	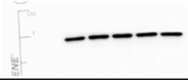	
p-c-jun	48 kDa	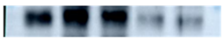	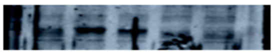
c-jun	48 kDa		
EGR1	75 kDa	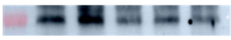	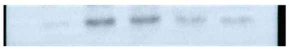
Actin	43 kDa	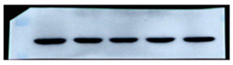	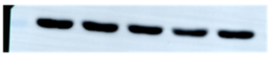

Figure S3. Raw data from western blot in Figure 5. The number of each independent experiment is indicated by #1 and #2.

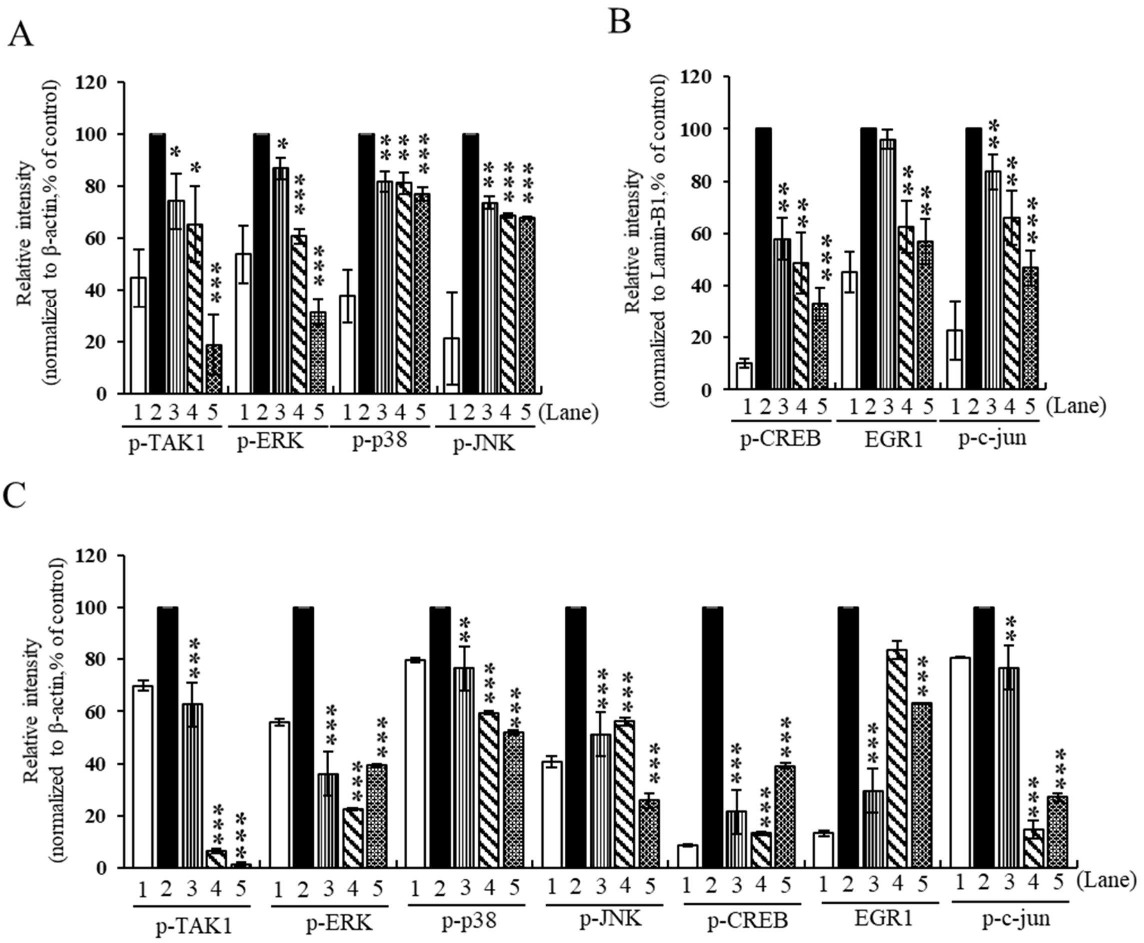

Figure S4. Quantification and Statistical Analysis of Western Blot in Figure 4. Bar graphs represent means ± S.D.. Densitometry calculations were performed using blots from two independent experiments (**p < 0.01, and ***p < 0.001 for comparison with controls, which were treated with PMA alone).

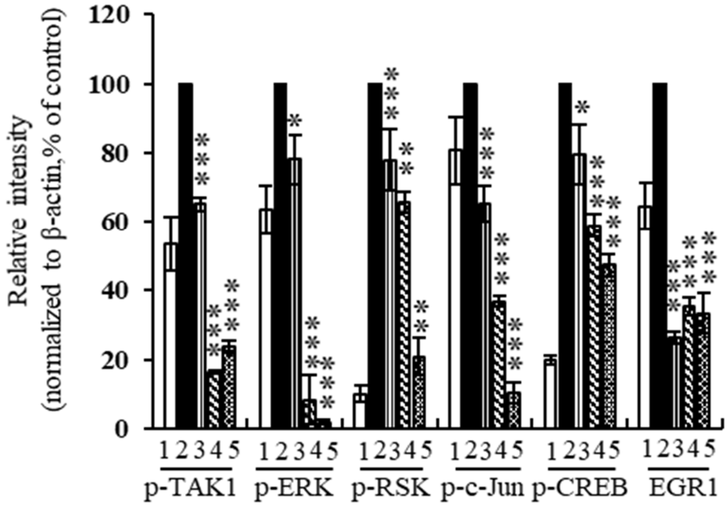

Figure S5. Quantification and Statistical Analysis of Western Blot in Figure 5. Densitometry calculation was done using the blots from two independent experiments. Bar graphs represent means ± S.D.. Densitometry calculations were performed using blots from two independent experiments (**p < 0.01, and ***p < 0.001 for comparison with controls, which were treated with PMA alone).
